# Dynamic Video Image Segmentation Based on Dual Channel Convolutional Kernel and Multi-Frame Feature Fusion

**DOI:** 10.3389/fnbot.2022.845858

**Published:** 2022-04-25

**Authors:** Zuguo Chen, Chaoyang Chen, Ming Lu

**Affiliations:** ^1^Shenzhen Institute of Advanced Technology, Chinese Academy of Sciences, Shenzhen, China; ^2^School of Information and Electrical Engineering, Hunan University of Science and Technology, Xiangtan, China; ^3^CAS Key Laboratory of Human-Machine Intelligence-Synergy Systems, Shenzhen, China

**Keywords:** dynamic video image segmentation, dual channel convolution kernel, multi-frame feature fusion, fire hole, aluminum electrolysis cell

## Abstract

The color image of the fire hole is key for the working condition identification of the aluminum electrolysis cell (AEC). However, the image of the fire hole is difficult for image segmentation due to the nonuniform distributed illuminated background and oblique beam radiation. Thus, a joint dual channel convolution kernel (DCCK) and multi-frame feature fusion (MFF) method is developed to achieve dynamic fire hole video image segmentation. Considering the invalid or extra texture disturbances in the edge feature images, the DCCK is used to select the effective edge features. Since the obtained edge features of the fire hole are not completely closed, the MFF algorithm is further applied to complement the missing portion of the edge. This method can assist to obtain the complete fire hole image of the AEC. The experiment results demonstrate that the proposed method has higher precision, recall rate, and lower boundary redundancy rate with well segmented image edge for the aid of working condition identification of the AEC.

## 1. Introduction

The aluminum electrolysis (AE) production process is a complex and continuous process, where any trivial failure in each step may affect the quality of the whole production process, resulting in poor uniformity of the products, low production efficiency, or extra energy resources consumption (Chen et al., 2021). It is known that the aluminum electrolysis cell (AEC) is the main production equipment in the AE production, which should be closely monitored online *via* the video as the currently adopted main measure. Then, image segmentation is the first essential dealt step to obtain visual features (Yue et al., 2020). The video image segmentation methods can be divided into the static feature and dynamic feature two categories (Bragantini et al., 2020).

The static features mainly include color, shape, contour, and texture. Wang proposes an Otsu image threshold segmentation method based on an improved particle swarm optimization (PSO) (Wang et al., 2019), where the inter-class variance of the Otsu is selected as the fitness function so as to increase the diversity of the particles with new particles supplement. Furthermore, a fast threshold image segmentation based on 2D fuzzy fisher and random local optimized quantum particle swarm optimization is proposed to reduce the redundant computation and improve the processing speed of the image segmentation (Zhang et al., 2016). Seyedhosseini and Tasdizen (2015) propose a semantic image segmentation based on the upper and lower hierarchical model to optimize the joint posterior probability. Dhanachandra and Chanu (2020) propose an image segmentation algorithm based on fuzzy c-means clustering algorithm (FCM) for noise image segmentation. However, it is rather difficult for static features to adapt to image segmentation with interference in complex scenes.

The dynamic features of the video images cannot be extracted with a single image but require to process continuous image frames consecutively in real-time. Karunanayake et al. (2020) has proposed a segmentation method based on multiple walking particles bouncing from the image edge to handling single or multiple objects characterized by a noisy background and broken boundaries. To balance the spatiotemporal coherence in scenes with deformation or large motion, a segmentation method is proposed based on the Markov chain model (Xixi and Chengmao, 2016). In terms of consecutive multi-frame images, an adaptive-domain network based on CycleGAN is proposed to improve the quality of the generated images in the feature space, making the translated images more informative for semantic segmentation (Cao et al., 2019). For moving object detection and segmentation, a feature extraction based adaptive neuro-fuzzy inference system (ANFIS) classifier is proposed (Guo et al., 2020), where the extracted features are trained and classified using the ANFIS classification module to improve the accuracy and recall rate of the image segmentation.

It is known that the fire hole images of the AEC are quite different under various working conditions, with high degree coupling between the target area and the background due to the background reflection, aerial fog, dust, and other interferences. Here, a dynamic video image segmentation method is proposed based on dual channel convolutional kernel (DCCK) and multi-frame feature fusion (MFF) to tackle these problems. The DCCK is proposed to select the effective edge features from the edge feature image. The core of the DCCK is a 4×4 convolution kernel which is used to obtain the edge image with smaller edges and interference texture. The other 6×6 convolutional kernel is used to obtain the salient edge with rough edges and interference texture. The multiplication of the two dealt results is applied to acquire the neat and smooth edge image. Since the edge features of the fire hole are not completely closed, an MFF algorithm is further developed to complement the missing portion by fusing two or more different frame images together.

The main contributions of the article are summarized as:

- A DCCK is proposed to select the effective edge features from the edge image *via* two different sized convolution kernels.
- A MFF method is developed to enhance the edge features and complement the missing edge portion by fusing more different frame images.
- Comparison experiments have been performed to demonstrate that the proposed method has higher mean pixel accuracy and lower boundary redundancy rate with satisfied image segmentation performance.

The remainder of the article is organized as follows. Section 2 presents the edge features obtainment based on the Prewitt operator and effective edge feature information selection based on DCCK. Section 3 develops a novel edge feature continuity processing algorithm based on MFF. Experiments are provided to verify the efficacy of the proposed method in Section 4. The conclusion is given in Section 5.

## 2. Video Image Segmentation of the Fire Hole

When the working condition of the AEC changes, the fire hole features of the AEC will change. Accordingly, the fire hole features are the key criteria to identify the work conditions, while image segmentation is the first essential dealt step to obtain visual features of the fire hole.

### 2.1. Edge Feature Obtainment

**Figure 1:**
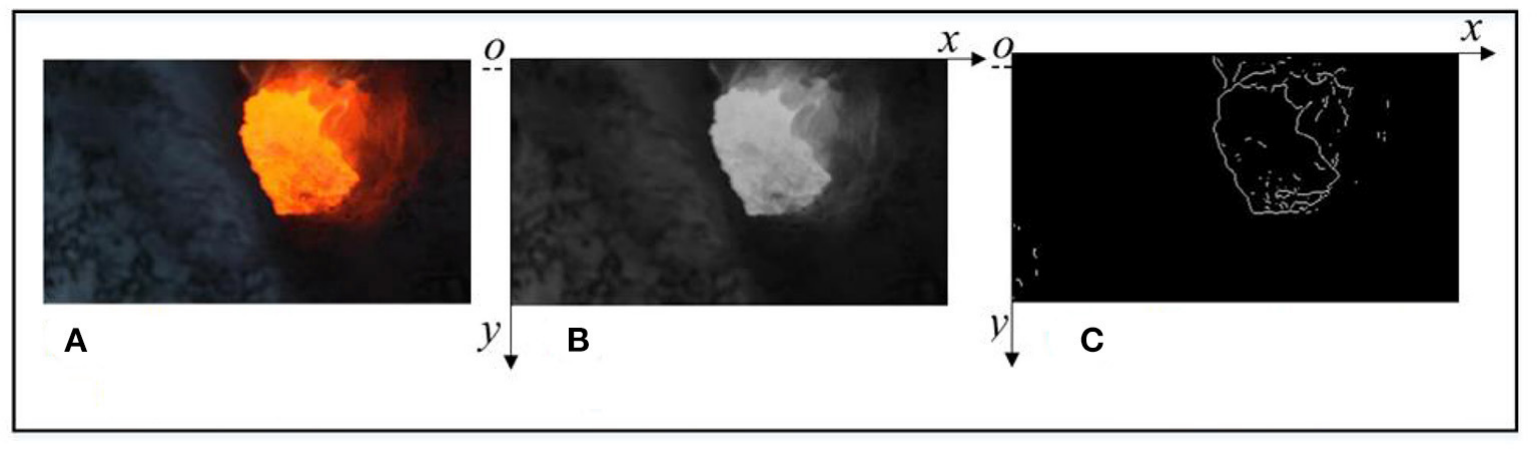
The obtained edge feature images (Prewitt operator). **(A)** The original image of the fire hole. **(B)** The gray image of the fire hole. **(C)** The obtained edge feature image.

The dealt image data are obtained from a 400KA aluminum electrolytic plant in Dengfeng City, Henan Province. The industrial cameras are installed to obtain the video data stream of the fire hole of the AEC. In order to obtain the object region in the fire hole image, edge features of the fire hole image should be extracted by the Prewitt operator before image segmentation. The RGB image, gray image, and the obtained edge feature image with the Prewitt operator of the fire hole are shown in Figure 1.

### 2.2. Effective Edge Features Selection Based on DCCK

The obtained edge feature image would contain a large number of invalid edge features of the fire hole. In order to acquire effective edge features of the fire hole, the convolutional neural network (CNN) is applied to deal with the edge features of the fire hole (Bui et al., 2019). With the different sizes of the convolutional kernels, the edge image can be dealt to connect the closer pixels and add the cover area of the edge. The sizes of the convolutional kernels are selected based on human experience with the trial and error method. In this article, a 4×4 convolutional kernel and another 6×6 convolutional kernel are selected to deal with the edge feature image, where the pixel values (*x, y*) at the convolutional kernels are set as *f*_1_(*x, y*) and *f*_2_(*x, y*). As seen on the left side of Equation (1), the selected 4×4 convolutional kernel *sk*_1_ is used to filter out the detailed images so as to connect scattered points of the image as much as possible. Moreover, the 6×6 convolution kernel *sk*_2_ is written on the right side of Equation (1),


(1) $$sk_{1}=\left[\begin{array}{cccc} 0 & 0 & 0 & 0\\ 0 & 1 & 1 & 0\\ 0 & 1 & 1 & 0\\ 0 & 0 & 0 & 0 \end{array}\right];\;sk_{2}=\left[\begin{array}{cccccc} 0 & 0 & 0 & 0 & 0 & 0\\ 0 & 1 & 1 & 1 & 1 & 0\\ 0 & 1 & 1 & 1 & 1 & 0\\ 0 & 1 & 1 & 1 & 1 & 0\\ 0 & 1 & 1 & 1 & 1 & 0\\ 0 & 0 & 0 & 0 & 0 & 0 \end{array}\right]$$

**Figure 2:**
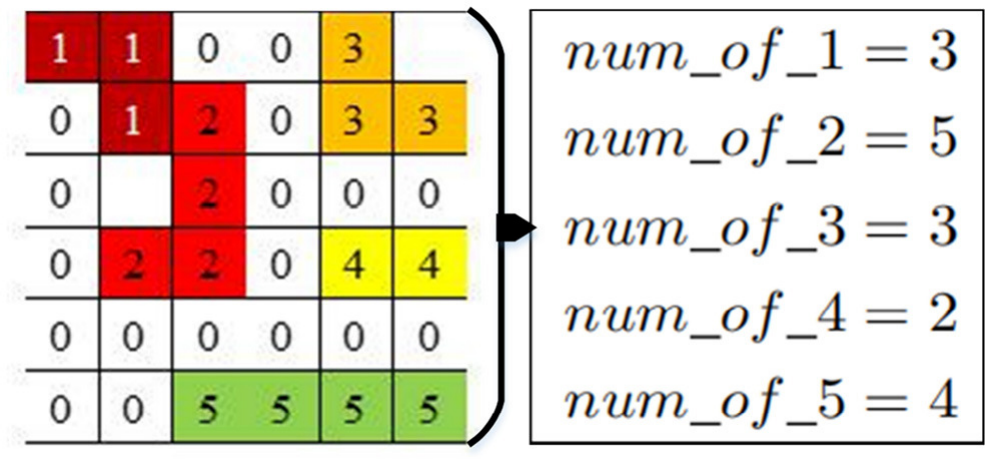
The image processed with different convolutional kernels.

For the purpose of expending edge width to enlarge the range of the boundary connection, such dual kernel operation can obtain more edge breakpoints to be closed or connected. After being processed with the 4×4 convolutional kernel of the edge image, the number of all continuous image blocks can be calculated, and each pixel in the block is labeled, as shown in the left graph of Figure 2.

**Figure 3:**
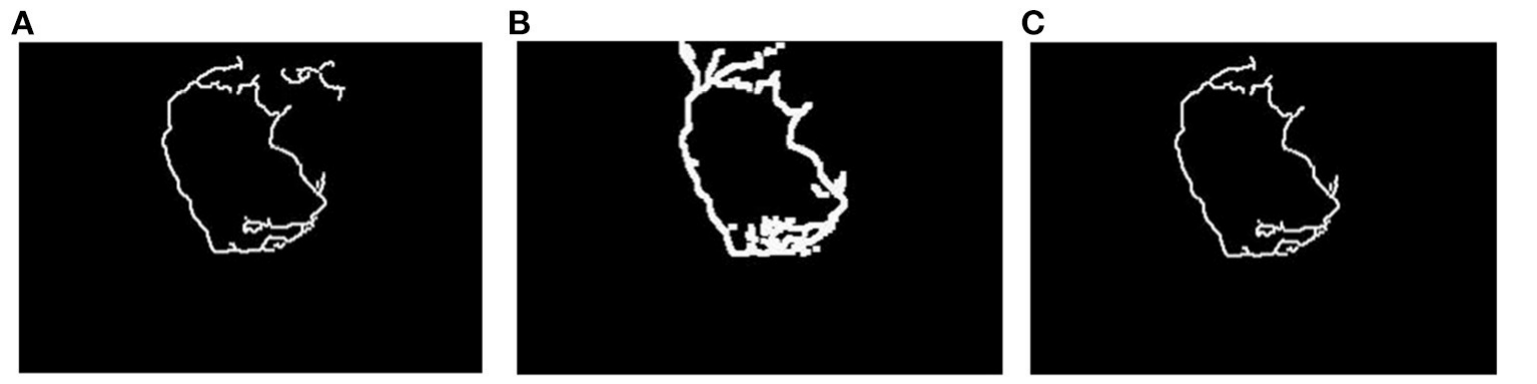
The illustration of the continuous pixel labeling. **(A)** The image processed with a 4 × 4 convolutional kernal. **(B)** The image processed with a 6 × 6 convolutional kernal. **(C)** The processed image with double channel convolutional kernal.

Next, the number of each non-zero digital tag is calculated in the continuous pixel labeling. In Figure 2, *num*_*of*_*i*, (*i* = 1, 2, ⋯ , 5) is the number of different digital tags in the box, which can easily obtain the number of pixels in each block, and removes pixel blocks with fewer pixels less than the *threshold*_1_ (set as *n*_*l*_) and turn their label as 0. After filtering, the unfiltered label is turned to 1 again (set as *N*_*g*_), written as,


(2) $$\begin{array}{l} {}[1]num\_of\_n_{l}\leq threshold_{1}\to f(x_{j},y_{j})=0\\ \;elsef(x_{k},y_{k})=1 \end{array}$$

where *f*(*x*_*j*_, *y*_*j*_) = *num*_*of*_*n*_*l*_ is the pixel number in the pixel block *n*_*l*_. *l* is the serial number of the pixel block; *j* is the serial number of the pixel point less than the *threshold*_1_. *N*_*g*_ is the label of the pixel block; *g* is the serial number of the pixel point greater than the *threshold*_1_. After processing by the 4×4 convolutional kernel, the obtained image is shown in Figure 3A. The image processing procedure *via* the 6×6 convolutional kernel is similar to that of the 4×4 convolutional kernel, and the processed image with the 6×6 convolutional kernel is shown in Figure 3B.

Afterward, the corresponding positions of the processed images with the 4×4 convolutional kernel *f*_1_(*x, y*) and the 6×6 convolutional kernel *f*_2_(*x, y*) are multiplied, where the multiplication process of the pixel values is shown in Table 1 and the resulted image is depicted in Figure 3C. It can be seen from Figure 3C that there is a relatively complete edge image containing little texture features. However, the edge is not completely enclosed for each frame image, which is unsuitable for the image obtainment of the fire hole. Hence, the edge feature continuity has to be further processed.

**Table 1:** The multiplication process of the pixel values.

***f*** **_1_(** ***x, y*** **)**	***f*** **_2_(** ***x, y*** **)**	***f*****_1_(*****x, y*****) ×** ***f*****_2_(*****x, y*****)**
0	0	0
0	1	0
1	0	0
1	1	1

## 3. Edge Feature Continuity Processing Based on MFF

**Figure 4:**
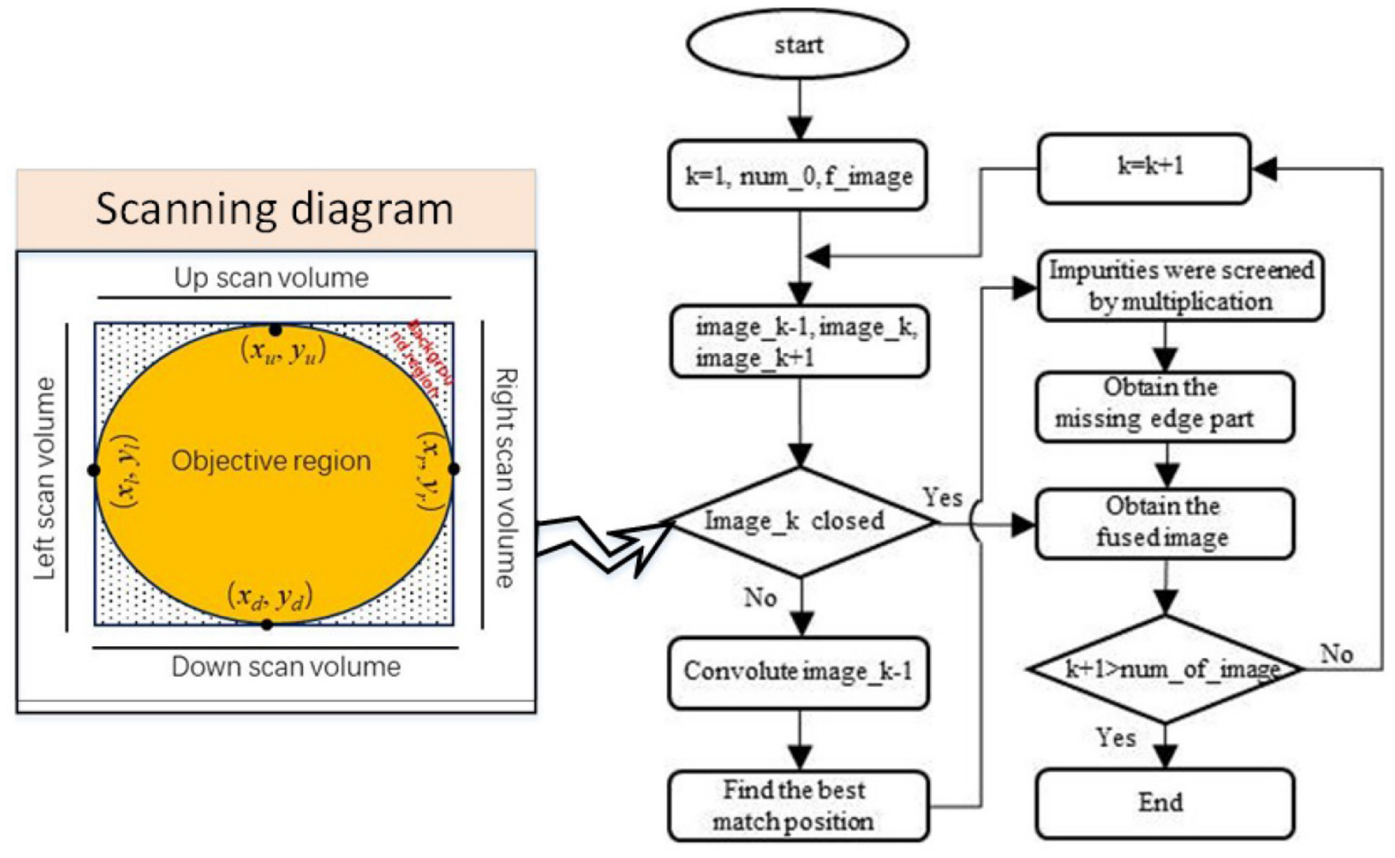
The flow chart of the edge feature continuity processing.

Since it is difficult to acquire a closed edge image for each frame image with large impurities to be filtered from only edge feature selection, an MFF method is proposed to complement the missing portion of the edge. The flow chart of the MFF method is illustrated in Figure 4.

**Figure 5:**
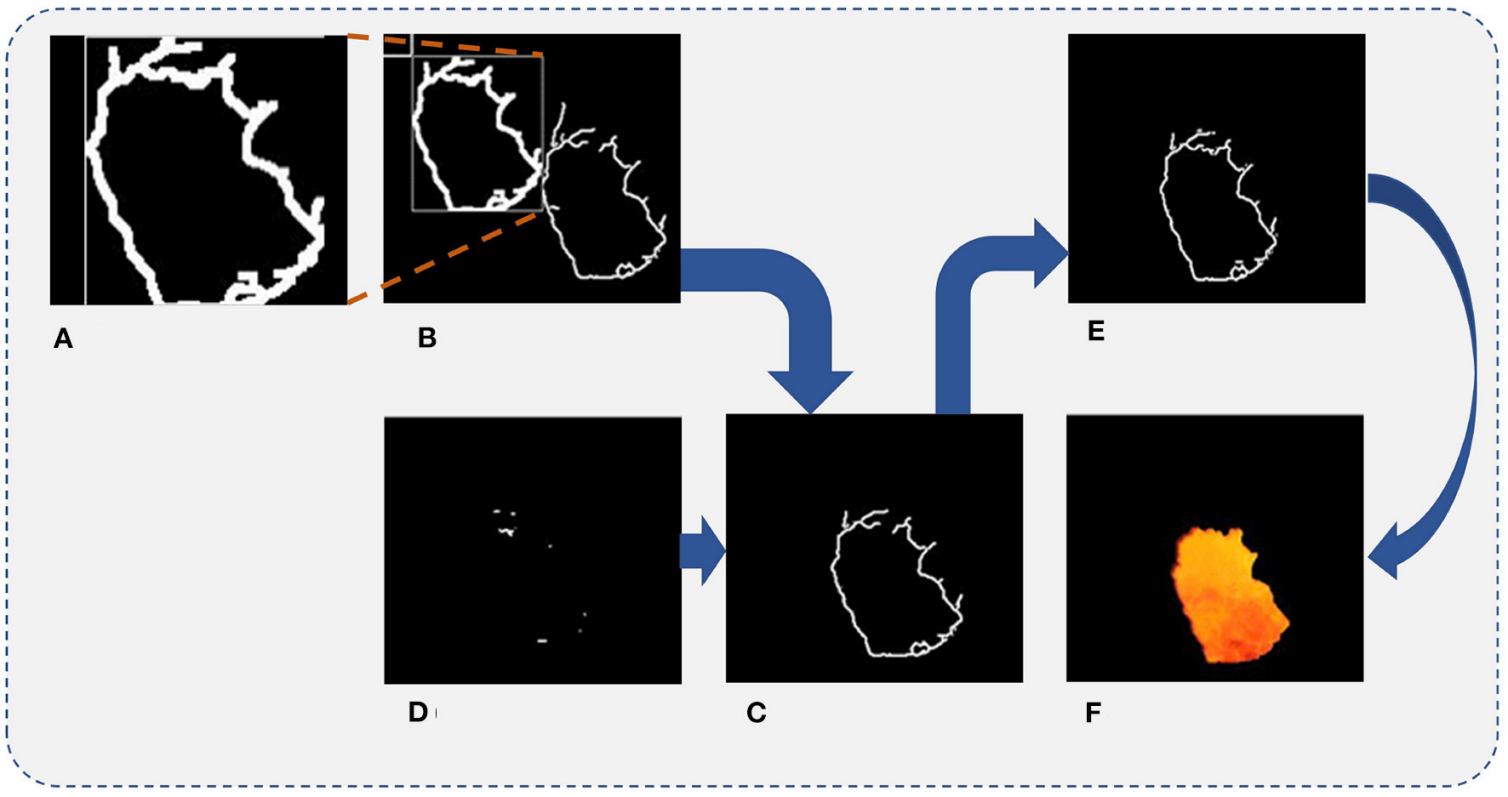
Final separated result of the fire hole. **(A)** The processed previous frame edge image. **(B)** Matching diagram of the edge image. **(C)** The overlapped part of the two images. **(D)** The missing part of the next frame image. **(E)** The continuous edge feature image. **(F)** Final separated result of the fire hole.

In order to determine whether the previous frame edge image has formed a complete edge pattern, a scanning algorithm (from four directions: up, down, left, and right) is adopted (refer to the left graph in Figure 4). If the edge feature image is closed, its label is marked as 1, and the frame is used as the reference edge feature image to mend the next frame of the edge feature image. Otherwise, the fused edge image of the previous frame with a complete edge pattern is used as the reference to complement the next frame edge image. When two frame edge images are fused, the previous frame of the edge image has first to be expanded by the convolutional operation to retain as many edge features as possible from the previous edge image (refer to Figure 5A).

The processed previous frame edge images by the convolutional operation are marked as *image*_(*k*−1)__*handled*. To find the optimal matching position, the *image*_(*k*−1)__*handled* edge image moves to the next frame edge image to find the optimal matching position of the next frame edge feature, the matching diagram is shown in Figure 5B. The object function is designed to determine the optimal matching position, written as,


(3) $$\begin{array}{l} max(\sum_{i=1}^{im_{\left(k-1\right)}\_r}\sum_{j=1}^{im_{\left(k-1\right)}\_c}part(i_{k-1},j_{k-1})\times part(i_{k},j_{k})\\ \;i=1,2,\cdots ,im_{\left(k-1\right)}\_r-im_{\left(k\right)}\_r\\ \;j=1,2,\cdots ,im_{\left(k-1\right)}\_c-im_{\left(k\right)}\_c \end{array}$$

where the *im*_(*k*−1)__*r* and *im*_(*k*−1)__*c* are the pixel row and column number of the *image*_(*k*−1)_. The *im*_(*k*)__*r* and *im*_(*k*)__*c* are the pixels row and column number of the next frame edge image. The *part*(*i*_*k*−1_, *j*_*k*−1_), *part*(*i*_*k*_, *j*_*k*_) are the pixel value of the *image*_(*k*−1)_ and the next frame edge image *image*_(*k*)_.

After obtaining the optimal matching position, the overlapped region of the two images is obtained by multiplying each pixel of the *image*_(*k*−1)_ and *image*_(*k*)_, while other positions are filled with 0. The image is marked as *Im*_*d*_(*k*)_, as shown in Figure 5C.

The *Im*_*d*_(*k*)_ is dealt with by the convolutional operator to extract the effective part for optimal matching by subtracting the pixels of the *Im*_*d*_(*k*)_ from the effective edge pixels at the corresponding position of *Im*_*d*_(*k*−1)_, and the corresponding subtraction relationship is shown in Table 2. Thus, the missing part of the *Im*_*d*_(*k*)_ is obtained and marked as *Im*_*sup*_(*k*)_, as shown in Figure 5D.

**Table 2:** The corresponding relationship of the substract processing.

***Im*** **_(***k***−1)_** **(***x, y***)**	***Im*** **_(***k***)_** **(***x, y***)**	**Substract**	**Standardization**
0	0	0	0
0	1	-1	0
1	0	1	1
1	1	0	0

Finally, the edge feature image of the *Im*_(*k*+1)_ is superimposed by adding the pixel values in the same position of the *Im*_*d*_(*k*)_ and the *Im*_*sup*_(*k*)_. The corresponding relationship of the superimpose processing is shown in Table 3, and the continuous edge feature image is shown in Figure 5E.

**Table 3:** The corresponding relationship of the superimpose processing.

***Im*** **_** ***d*** **_(***k***)_** **(***x, y***)**	***Im*** **_** ***sup*** **_(***k***)_** **(***x, y***)**	**Superimpose result**	**Standardization**
0	0	0	0
0	1	1	1
1	0	1	1
1	1	2	1

In order to fill the image, the interior of the edge feature should be completely filled using the white color, and the two-dimensional binary image can be converted into a three-dimensional RGB image (Guan et al., 2019). To remain the color feature of the separated fire image as the same as the original image, the three-dimensional RGB image is merged with the original image, as shown in Figure 5F. It can be seen from the final dealt result that the dynamic video image segmentation method based on DCCK and MFF can effectively segment the image and obtain a completely smooth edge image without much impurity interference.

## 4. Experimental Results and Analysis

Three indicators(precision, recall rate, and F1-Measure) are used to evaluate the segmentation effect, and precision represents the proportion of samples identified as positive categories that are indeed positive categories. Recall rate represents the proportion of all positive class samples that are correctly identified. F1-Measure represents the harmonic average evaluation index of precision and recall rate.

The Roberts operator (Albdour and Zanoon, 2020) detects edge lines by local difference calculation and is often used to process low-noise images with steepness. The edge localization effect of the Sobel operator (Zhou and Liu, 2019) is good, and it is better for image processing with more noise, but the detected edge is prone to multi-pixel width. The Prewitt operator (Song et al., 2019) has a better effect on the image edge extraction of grayscale gradient and does not consider the influence of the distance of adjacent points on the current pixel point. Compared with other classical edge detection operators, the Canny operator (Bu et al., 2019) has higher accuracy, detects finer edges and requires more computation, and is the most representative edge detection operator.

**Figure 6:**
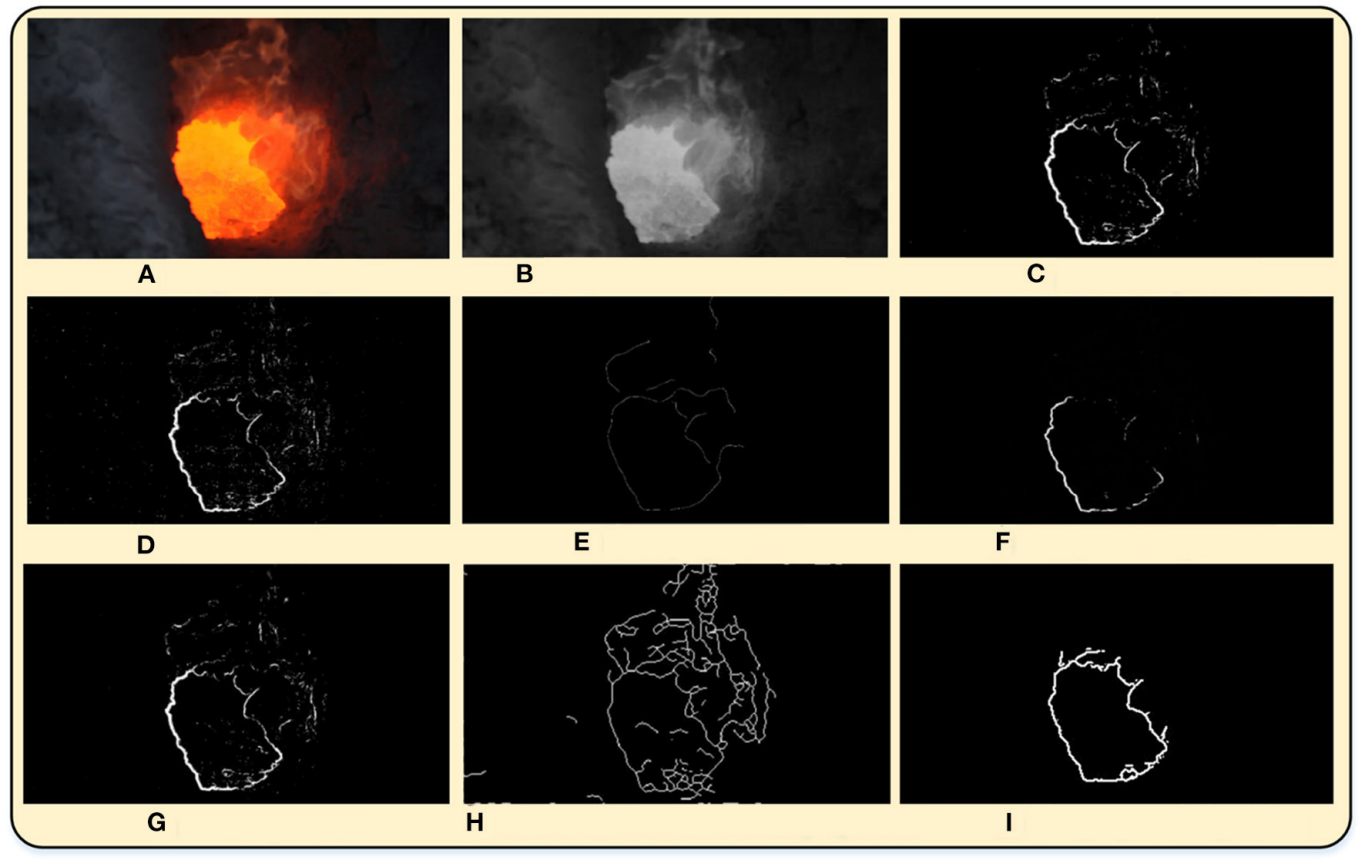
The segmentation results with different algorithms for comparison. **(A)** Original image. **(B)** Gray-scale map image. **(C)** Prewitt operator. **(D)** Roberts operator. **(E)** Canny operator. **(F)** Funny-sobel operator. **(G)** Sobel operator. **(H)** Bilateral filter based Canny operator. **(I)** The proposed algorithm.

In order to verify the effectiveness of the proposed method, the Roberts operator, Prewitt operator, Sobel operator, Canny operator, fuzzy-Sobel operator (Sivaranjani and Kalaiselvi, 2021), and bilateral filter based Canny operator (Zhang et al., 2019) are used for comparison. The experimental results are shown in Figure 6, and it can be seen that most methods are unable to segment the closed image edge completely.

When the Canny operator is used to obtain the edge images of the fire hole, most of the noise can be removed, but the obtained edge images are discontinuous with less remarkable edge lines. The fuzzy Sobel operator can filter out most of the noise of the edge images and the dust textures, however, the edge images of the fire hole still have a lot of missing parts. While the bilateral filtering based Canny operator can obtain the complete edge images, many invalid textures still remain. As the proposed DCCK joint MFF method can not only remove most of the noise of the edge images of the fire hole but also can obtain continuous and complete edge images of the fire hole. Therefore, the performance of the proposed method is significantly higher than those of traditional algorithms in the processing of the segmentation of the fire-hole of the AEC. The evaluation indexes (precision, recall rate, F1-Measure) of the image segmentation are calculated as well, listed in Table 4. The recall rate and F1-Measure of the obtained edge image using the proposed algorithm are the best among these algorithms. Although the precision of the obtained edge image with the proposed algorithm is slightly lower than that of the Prewitt and Sobel operator, it can be ignored without causing performance degradation in practice. In all, the proposed algorithm ranks highest comprehensively compared with other image segmentation algorithms. Compared with other methods of image segmentation, the algorithm of this article has a remarkable improvement in applicability and general utilization.

**Table 4:** The evaluation indexes of the image segmentation with different algorithms comparison.

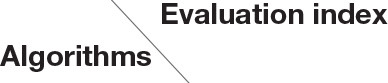	**Precision**	**Recall rate**	**F1-Measure**
Canny operator	0.0429	0.0598	0.0179
Bilateral filter based Canny operator	0.1676	0.2851	0.8554
Fuzzy sobel operator	0.4151	0.6530	1.9591
Roberts operator	0.4430	0.5464	1.6391
Prewitt operator	0.5363	0.5962	1.7886
Sobel operator	**0.6102**	0.5613	1.6839
The proposed algorithm	0.5132	**0.8554**	**2.5663**

*Bold value means that these values are the best*.

## 5. Conclusion

This article proposes a dynamic video image segmentation method based on the DCCK joint MFF algorithms to segment the images of the fire hole of the AEC. The Prewitt operator is first used to extract the edge features of the fire image. Due to the extra texture in the edge feature image, the DCCK is proposed to select the effective edge features. Then the MFF algorithm is further proposed to complement the missing portion of the edge image. Finally, the performance of the proposed method is verified with a comparison of other segmentation methods in dealing with images of the fire hole AEC under heavy dust and complex background interference. Compared with the conventional method of image segmentation, the proposed method has high precision with wide applicability.

## Data Availability Statement

The original contributions presented in the study are included in the article/supplementary material, further inquiries can be directed to the corresponding author/s.

## Author Contributions

ZC determined the research program and writed the article. The experimental data were analyzed by CC. The theory of the model was given and analyzed by ML. All authors contributed to the article and approved the submitted version.

## Funding

The research has been supported by grants from the China Postdoctoral Science Foundation (Ref. 2020M672890), the National Natural Science Foundation of China (Ref. 61903137), the Natural Science Foundation Of Hunan Province (Ref. 2020JJ5201), and the Shenzhen Basic Research Program (Ref. Jcyj20170818153635759).

## Conflict of Interest

The authors declare that the research was conducted in the absence of any commercial or financial relationships that could be construed as a potential conflict of interest.

## Publisher's Note

All claims expressed in this article are solely those of the authors and do not necessarily represent those of their affiliated organizations, or those of the publisher, the editors and the reviewers. Any product that may be evaluated in this article, or claim that may be made by its manufacturer, is not guaranteed or endorsed by the publisher.
